# Understanding Associations Between Patient‐Level Factors and Participation in a Breast Cancer Clinical Trial

**DOI:** 10.1002/cam4.71010

**Published:** 2025-08-20

**Authors:** Nicole E. Caston, Luqin Deng, Courtney P. Williams, Emily B. Levitan, Andres Azuero, Russell Griffin, Karen L. Smith, Antonio C. Wolff, Michelle E. Melisko, Eileen H. Shinn, Kathleen Gallagher, Rebekah Angove, Stephanie B. Wheeler, Gabrielle B. Rocque

**Affiliations:** ^1^ Division of Hematology and Oncology, Department of Medicine University of Alabama at Birmingham (UAB) Birmingham Alabama USA; ^2^ Department of Epidemiology UAB Birmingham Alabama USA; ^3^ Division of Preventive Medicine UAB Birmingham Alabama USA; ^4^ School of Nursing UAB Birmingham Alabama USA; ^5^ Merck Rahway New Jersey USA; ^6^ Johns Hopkins University Sidney Kimmel Cancer Center Baltimore Maryland USA; ^7^ Division of Hematology and Oncology, Department of Medicine University of California San Francisco San Francisco California USA; ^8^ Department of Behavioral Science University of Texas MD Anderson Cancer Center Houston Texas USA; ^9^ Patient Advocate Foundation Hampton Virginia USA; ^10^ Department of Health Policy and Management, Gillings School of Global Public Health University of North Carolina at Chapel Hill Chapel Hill North Carolina USA; ^11^ University of North Carolina at Chapel Hill Lineberger Comprehensive Cancer Center Chapel Hill North Carolina USA; ^12^ Division of Gerontology, Geriatrics, and Palliative Care UAB Birmingham Alabama USA; ^13^ O'Neal Comprehensive Cancer Center, UAB Birmingham Alabama USA

**Keywords:** attitudes toward trials, breast cancer, cancer clinical trials, clinical trial knowledge, vulnerable populations

## Abstract

**Background:**

Although breast cancer (BC) clinical trials offer novel treatments, participating patients often do not represent populations seen in clinics. This study assessed how patient sociodemographics, attitudes, and knowledge about clinical trials may be associated with participation.

**Methods:**

This cross sectional analysis used survey data collected August–September 2021 by Translational Breast Cancer Research Consortium (TBCRC) and December 2022 by Patient Advocate Foundation (PAF) among women with a BC diagnosis. Respondents reported BC clinical trial participation, Attitudes Toward Cancer Trials Scale, clinical trial knowledge, diagnosis age, race, education level, household income, employment status, and BC stage. Descriptive and bivariate analyses were conducted using Cramer's *V* or Cohen's *d* as effect sizes. Standardized total effects (*b*_Tot_) were estimated using a prespecified Structural Equation Model with 0.1, 0.3, and 0.5 indicating weak, medium, and large magnitude, respectively.

**Results:**

Of 612 respondents, 26% were Black, Indigenous, or a Person of Color, 44% < Bachelor's degree, and 48% had incomes < $50,000. Respondents who reported trial participation (18%) more often had positive attitudes toward trials (mean 94 of 126, SD 19.3 vs. 86, SD 15.5; *d* = 0.48) compared to those not reporting participation. Positive attitude was associated with trial participation for both survey cohorts (TBCRC: *b*_Tot_ = 0.22, *p* = 0.0002; PAF: *b*_Tot_ = 0.13, *p* = 0.01). Furthermore, clinical trial knowledge was associated with attitudes (TBCRC: *b*_Tot_ = 0.27, *p* = < 0.0001; PAF: *b*_Tot_ = 0.15, *p* = 0.003).

**Discussion:**

Although we found that positive attitudes and high knowledge were associated with clinical trial participation, it is unknown if the trial itself directly affected this. However, trial enrollment may be increased and diversified via future interventions focused on promotion of positive attitudes toward clinical trials, potentially through increased trial education and knowledge.

## 1 Background

Cancer clinical trial participants are often not representative of real‐world populations in clinical practice [1, 2]. In our previous study, we found 59% of women diagnosed with breast cancer were eligible to participate in a breast cancer clinical trial (patients were ineligible due to abnormal lab values, comorbidities, cancer‐specific criteria); however, 52% of those eligible were offered a trial. Furthermore, of those eligible and offered a trial, only 26% enroll onto a clinical trial [3]. Previous studies have found that patients who do not typically enroll on cancer clinical trials are Black or African American, older than 69 years of age, rural residents, individuals who have lower educational attainment, and those with lower annual incomes [3, 4, 5, 6, 7]. The abovementioned socioeconomic demographics are mostly non‐modifiable (race, age, residence, education) and therefore, interventions targeting demographics may not be the most effective way to increase clinical trial participation.

However, an individual's attitudes and knowledge about clinical trials may affect their participation. Past research showed that individuals rely on attitudes at the expense of deliberative consideration of a decision's risks and benefits, unless sufficient motivation and opportunity to think things through are present [8, 9, 10]. It is unknown how attitudes and knowledge influence actual clinical trial participation, especially as these factors are modifiable. Furthermore, the interplay between patient‐level demographics and attitudes toward clinical trials is important as different patient populations may have varying attitudes and therefore specific interventions may be needed to target specific groups.

This study aimed to understand which patient‐level factors—including both non‐modifiable and moldable factors—were associated with breast cancer clinical trial participation among two survey cohorts of women with breast cancer, including those typically represented and underrepresented in cancer clinical trials. Furthermore, we sought to better understand the relationship between patient‐level demographics and attitudes toward clinical trials.

## 2 Methods

### 2.1 Study Design and Participants

This cross sectional study used secondary survey data from two nationwide cohorts of female respondents with self‐reported breast cancer who received treatment from either academic or community hospitals. The survey was created by Translational Breast Cancer Research Consortium (TBCRC) investigators (Figure S1) to understand how COVID‐19 related anxiety affected willingness to participate in a clinical trial, along with understanding willingness to participate in pandemic‐era modified trials (Johns Hopkins Institutional Review Board (IRB00286085); TBCRC 057 study) [11]. This survey was fielded from August through September 2021 via social media posts containing promotional materials with the survey link by breast cancer advocacy organizations and TBCRC investigators. The advocacy groups promoted the survey through their online platforms, and the TBCRC investigators were encouraged to promote the survey on their personal platforms. The survey was also fielded in December 2022 to women with breast cancer recently served by Patient Advocate Foundation (PAF). PAF is a nationwide nonprofit organization that provides social needs navigation and financial aid to patients diagnosed with a chronic illness. Patients seeking services from PAF face healthcare access and affordability challenges, with many representing marginalized populations who face a variety of social needs and are limited in available resources to meet both medical and nonmedical needs [12]. Inclusion criteria for this study included female sex with a diagnosis of breast cancer at any stage within the last 5 years or metastatic breast cancer at any time, the ability to complete the survey in English, ≥ 18 years of age, and US resident. The PAF survey required all survey questions to be answered to ensure complete data capture. To optimize representation of individuals typically underrepresented in clinical trials, PAF sent additional survey reminders to those living in higher disadvantaged areas and who had previously self‐reported their race as Black, Indigenous, or a Person of Color (BIPOC). The current study was approved by the University of Alabama at Birmingham Institutional Review Board (IRB‐300010015). By beginning the survey, respondents provided consent to participate in this research.

### 2.2 Outcome

#### 2.2.1 Participation in a Clinical Trial

Respondents were asked to answer yes or no to the question, “Have you ever participated in a breast cancer clinical trial?” In our questionnaire, we informed respondents that, “breast cancer clinical trials are a type of research done to learn about breast cancer and about how to improve breast cancer treatments.” Furthermore, breast cancer clinical trials involve individuals who have a diagnosis of breast cancer and typically follow these individuals throughout a course of treatment (whether they are in a control arm or the research arm; the control arm still receives standard of care treatment) to understand how the new treatment offers better care compared to the current standard. Along with new treatments, trials can test new screening technologies or interventions that increase quality of life [13].

### 2.3 Variables of Interest for Structural Equation Modeling

#### 2.3.1 Education Level

Highest level of education completed was self‐reported by survey respondents and grouped into high school diploma or less (less than high school, high school); some college (some college, Associate's degree); and Bachelor's degree or more (Bachelor's, Master's, Doctorate degree). For Structural Equation Modeling (SEM), years of education were used as follows: 9 years (less than high school), 12 (high school), 13 (some college), 14 (Associate's degree), 16 (Bachelor's degree), and 18 (Master's or Doctorate degree).

#### 2.3.2 Annual Household Income

Respondents reported their annual income in six categories (< $20,000, $20,000–$34,999, $35,000–$49,999, $50,000–$74,999, 75,000–$99,999, and more than $100,000). We then grouped income into < $50,000 and ≥ $50,000. For SEM, the median income of the reported category was used ($10,000, $28,000, $43,000, $58,000, $83,000, $110,000).

#### 2.3.3 Employment Status

Employment status was grouped as employed (working either part or full time [32 h or more/week]), unemployed/other (unemployed, temporarily laid off, do not work, student, other), or retired/disabled (retired, on short‐ or long‐term disability, permanently disabled). We grouped retired and disabled together, as these individuals were both receiving some form of income.

#### 2.3.4 Attitudes Toward Cancer Trials Scale

Using an 18‐item questionnaire developed by Schuber and colleagues, we assessed individual attitudes toward clinical trials [14]. Responses are on a 7‐point Likert scale (strongly disagree, disagree, somewhat disagree, neither agree nor disagree, somewhat agree, agree, strongly agree). Negatively worded items were reverse coded for scoring. Scores range from 18 to 126, with higher scores indicating more positive attitudes. We also dichotomized the scale into positive or negative‐leaning attitudes using the midpoint of responses. Four subscales were also measured. The (1) personal beliefs and (2) trust in the research process subscale scores ranged from 4 to 28; the (3) personal barriers and safety and (4) personal and social value subscale scores ranged from 5 to 35.

#### 2.3.5 Knowledge About Clinical Trials

A 7‐item questionnaire developed by Ellis and colleagues assessed clinical trial knowledge. Scores ranged from 0 to 7, with higher scores indicating greater clinical trial knowledge [15, 16]. We dichotomized scores into more (scores ≥ 5) vs. less knowledge (score ≤ 4). We chose the score of 4 because a score of 4/7 would be considered a failing grade (57% of correct answers). Response options included yes, no, don't know. If a respondent answered don't know, the question was marked as an incorrect answer.

#### 2.3.6 Cancer Stage

Cancer stage was self‐reported by survey respondents and grouped as early (with or without lymph node involvement) or late (having spread to other parts of the body).

### 2.4 Additional Variables of Interest

Respondents self‐reported all other variables of interest, including age at diagnosis (≤ 49 years, ≥ 50), race (White/Caucasian, Black/African American, American Indian/Alaska Native, Asian, Native Hawaiian or Pacific Islander, more than one race, other race), ethnicity (Hispanic or Latino or non‐Hispanic or Latino), years since cancer diagnosis (< 5, 5–10, > 10), and insurance status (private, Medicare, Medicaid, none/other/unknown). Respondents were also asked if they had ever had a discussion about clinical trials with a breast cancer provider (yes, no).

### 2.5 Statistical Analysis

Descriptive statistics were calculated using frequencies and percentages for categorical variables and means and standard deviations (SD) for continuous variables. Differences in characteristics for respondents who had and had not participated were calculated using measures of effect size, such as Cohen's *d* for numerical characteristics (i.e., the standardized mean difference; small: 0.2, medium: 0.5, large: 0.8) or Cramer's *V* for cross‐tabulations. *V* of 0.1 is considered a small effect, 0.3 a medium effect, and 0.5 a large effect when comparing across two categories; 0.1 a small effect, 0.25 a medium effect, and 0.4 a large effect when comparing across more than two categories [17]. We used SEM to fit a prespecified path model for patient characteristics as they influence clinical trial enrollment by survey cohort. We determined our prespecified path model based on a priori knowledge and hypotheses. We estimated our prespecified SEM model and evaluated fit using an estimator robust to non‐normality (direct robust method) [18]. Appropriate model fit was determined using the Root Mean Squared Error of Approximation (RMSEA) index. Values < 0.06 indicate good model fit, values from 0.06 to 0.1 indicate fair fit, and values > 0.1 indicate poor fit [19, 20]. We ran an overall test of equality of regression paths across survey cohorts to determine if the SEM should be split by survey cohorts using Chi‐Square difference and the corresponding *p* value. The null hypothesis is that the regression paths are similar. Using the estimated path coefficients, standardized direct, indirect, and total effects were estimated on the likelihood of clinical trial enrollment. The standardized path coefficients and effects were interpreted as standardized regression coefficients (0.1 weak, 0.3 medium, 0.5 large associations). A sensitivity analysis was performed including only respondents who reported discussing a clinical trial with their breast cancer provider.

We fitted an exploratory logistic regression model estimating odds ratios (OR), predicted probabilities, and 95% confidence intervals (CI) assessing the interaction between individual clinical trial attitudes and knowledge on having ever participated in a breast cancer clinical trial. This model controlled for age at diagnosis, race (due to small numbers, categories with < 20 individuals were grouped into a singular group for modeling purposes), ethnicity, cancer stage, education, employment, income, and survey cohort. We fitted an exploratory logistic regression model examining the association between patient‐level demographics and positive‐leaning attitudes toward clinical trials. Finally, we fitted an additional exploratory logistic regression model examining the association between patient‐level demographics and high trial knowledge. The statistical significance level was set to 0.05. Analyses were performed using SAS software, version 9.4 (SAS Institute, Cary, NC). PROC CALIS was used for fitting the path model. Overall test of equality of regression paths across survey cohorts was performed using R software, version 4.3.2.

## 3 Results

### 3.1 Sample Characteristics

Of 636 eligible female breast cancer respondents, 612 respondents were included (*n* = 254 TBCRC; *n* = 358 PAF). Due to missing data, 24 TBCRC respondents were excluded. Overall, 26% of respondents were BIPOC, 44% were not college educated, 48% had annual household incomes of < $50,000, and 44% were retired or disabled (Table 1). Compared to the PAF survey cohort, the TBCRC survey cohort was more often White (92% vs. 61%, *V* = 0.37), college educated (80% vs. 38% college educated, *V* = 0.43), had higher incomes (83% vs. 31% ≥ $50,000, *V* = 0.52), and employed (54% vs. 37%, *V* = 0.17; Table S1).

**TABLE 1:** Patient demographics and clinical characteristics of overall sample (*N* = 612), by participation in a breast cancer clinical trial (*n* = 109), or not (*n* = 503).

	Overall *N* = 612	Participated in a breast cancer clinical trial *n* = 109	Never participated in a breast cancer clinical trial *n* = 503	Cramer's *V* or Cohen's *d*
	*n* (%)			
Age at diagnosis, in years				
49 or younger	342 (56)	70 (64)	272 (54)	0.08
50 or older	270 (44)	39 (36)	231 (46)	
Race				
American Indian/Alaska Native	1 (0)	0 (0)	1 (0)	0.08
Asian	13 (2)	1 (1)	12 (2)	
Black/African American	109 (18)	19 (17)	90 (18)	
More than one race	18 (3)	3 (3)	15 (3)	
Native Hawaiian or Pacific Islander	1 (0)	0 (0)	1 (0)	
Other	19 (3)	1 (1)	18 (4)	
White/Caucasian	451 (74)	85 (78)	366 (73)	
Ethnicity				
Hispanic or Latino(a) origin or descent	50 (8)	8 (7)	42 (8)	0.01
Non‐Hispanic or Latino(a) origin or descent	562 (92)	101 (93)	461 (92)	
Education level				
High school or less	72 (12)	5 (5)	67 (13)	0.12
Some college	200 (33)	32 (29)	168 (33)	
Bachelor's degree or more	340 (56)	72 (66)	268 (53)	
Annual household income				
Less than $50,000	291 (48)	41 (38)	250 (50)	0.09
$50,000 or more	321 (52)	68 (62)	253 (50)	
Employment status				
Employed	268 (44)	48 (44)	220 (44)	0.06
Retired/disabled	269 (44)	52 (48)	217 (43)	
Unemployed/Other	75 (12)	9 (8)	66 (13)	
Breast cancer stage				
Early	315 (51)	42 (39)	273 (54)	0.12
Late	297 (49)	67 (61)	230 (46)	
Years since cancer diagnosis				
Less than 5 years ago	436 (71)	66 (61)	370 (74)	0.12
5–10 years ago	91 (15)	19 (17)	72 (14)	
More than 10 years ago	85 (14)	24 (22)	61 (12)	
Insurance				
Ever Medicaid	107 (17)	17 (16)	90 (18)	0.04
Ever Medicare	167 (27)	27 (25)	140 (28)	
None/other/unknown	42 (7)	9 (8)	33 (7)	
Private	296 (48)	56 (51)	240 (48)	
Breast cancer provider discussed a breast cancer clinical trial				
Yes	218 (36)	105 (96)	113 (22)	0.59
No	394 (64)	4 (4)	390 (78)	
Clinical trial knowledge score, mean (SD)	4.1 (2.0)	4.6 (1.7)	4.0 (2.0)	0.48
Clinical trial knowledge categorized				
High knowledge	291 (48)	66 (61)	225 (45)	0.12
Low knowledge	321 (52)	43 (39)	278 (55)	
Attitudes Toward Cancer Trials Scale, mean (SD)	87.3 (16.5)	93.7 (19.3)	85.9 (15.5)	0.48
Personal beliefs subscale score, mean (SD)	15.2 (6.0)	15.8 (6.5)	15.1 (5.9)	0.11
Personal barriers and safety subscale score, mean (SD)^a^	17.5 (6.7)	15.4 (6.7)	17.9 (6.6)	0.39
Personal and social value subscale score, mean (SD)	28.4 (6.3)	30.4 (7.0)	28.0 (6.0)	0.39
Trust in the research process subscale score, mean (SD)	21.1 (5.3)	23.0 (5.7)	20.7 (5.2)	0.43
Attitudes Toward Cancer Trials Scale categorized				
Positive‐leaning attitudes	503 (82)	100 (92)	403 (80)	0.12
Negative‐leaning attitudes	109 (18)	9 (8)	100 (20)	
Survey cohort				
Patient Advocate Foundation	358 (58)	54 (50)	304 (60)	0.08
Translational Breast Cancer Research Consortium	254 (42)	55 (50)	199 (40)	

*Note:* Clinical trial knowledge score ranges from 0 to 7 with higher scores indicating more knowledge about clinical trials. Knowledge scores equal to or > 5 were considered high knowledge, as this would be considered a passing grade. Attitudes Toward Cancer Trials Scale ranges from 18 to 126 with higher scores indicating more positive attitudes toward clinical trials. Personal beliefs and trust in the research process subscales range from 4 to 28; personal barriers and safety and personal and social value subscales range from 5 to 35.

^a^ Personal barriers and safety score is kept negative coded for subscale but reverse coded for overall score SD = standard deviation.

Overall, 18% of respondents had participated in a breast cancer clinical trial, which was similar across the two survey cohorts (22% TBCRC, 15% PAF). Compared to those who had not participated, respondents who participated in a clinical trial were more often college educated (66% vs. 53% had a Bachelor's degree or higher, *V* = 0.12) and had household income > $50,000 (62% vs. 50%, *V* = 0.09). Among individuals who did not participate in a breast cancer clinical trial, 22% (*n* = 113) reported having a clinical trial discussion with a provider.

Though overall clinical trial knowledge was low (mean score 4.1 of 7 [SD 2.0]), respondent attitudes toward clinical trials were mostly positive leaning (82%). Compared to those who had not, respondents who had participated in a clinical trial had higher mean knowledge (4.6 [SD 1.7] vs. 4.0 [SD 2.0]; *d* = 0.48) and more positive‐leaning attitudes (92% vs. 80%; *V* = 0.12).

### 3.2 Associations Between Demographics and Breast Cancer Clinical Trial Participation

**FIGURE 1:**
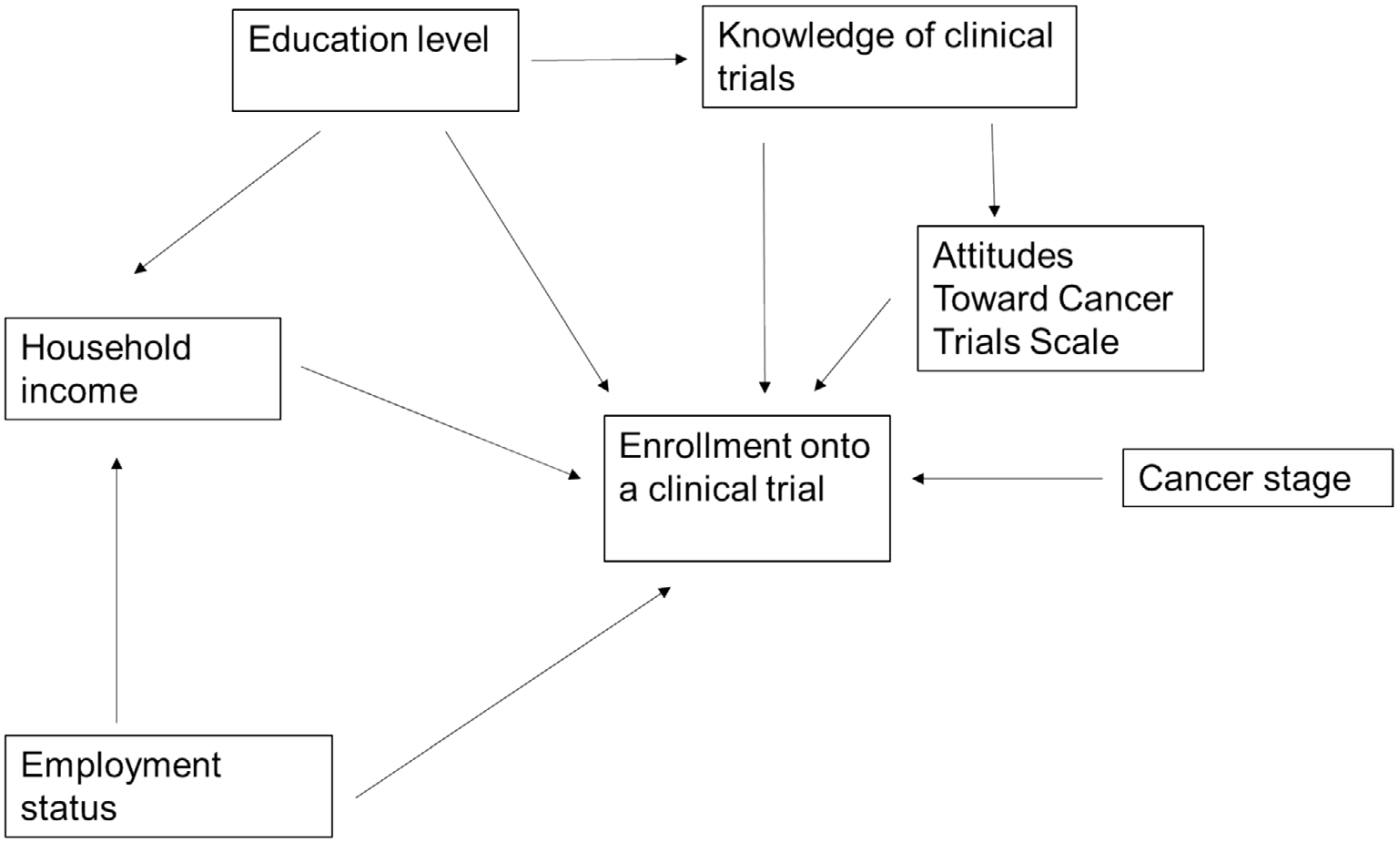
Hypothesized conceptual model.

**FIGURE 2:**
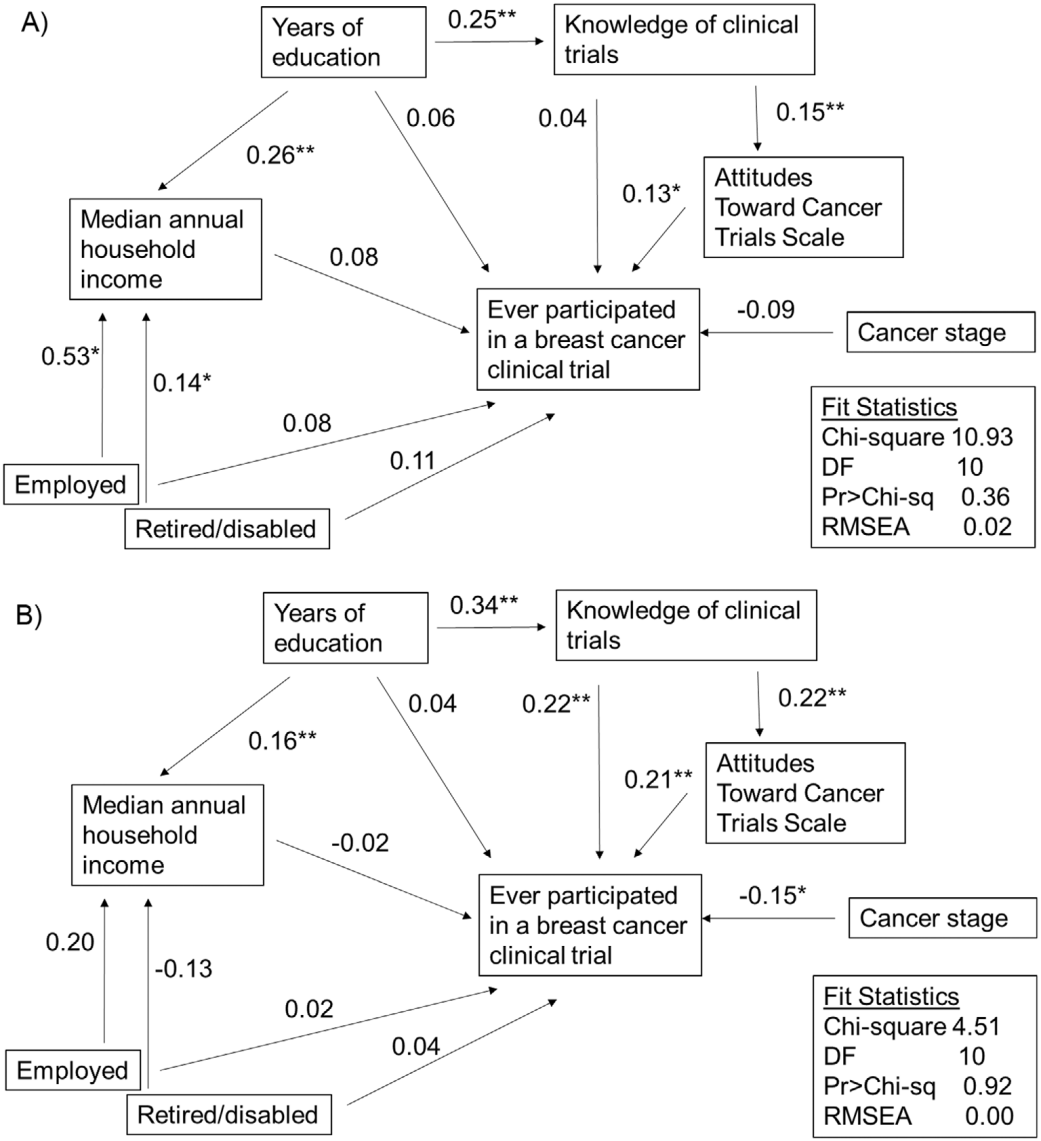
Final conceptual model with standardized direct effects by survey cohort: (A) Patient Advocate Foundation (*n* = 358) and (B) Translational Breast Cancer Research Consortium (*n* = 254). *p value ≤ 0.05; **p value ≤ 0.01. RMSEA values < 0.06 indicate good fit. Clinical trial knowledge score ranges from 0 to 7 with higher scores indicating more knowledge about clinical trials. Knowledge scores equal to or > 5 were considered high knowledge, as this would be considered a passing grade. Attitudes Toward Cancer Trials Scale ranges from 18 to 126 with higher scores indicating more positive attitudes toward clinical trials. Personal beliefs and trust in the research process subscales range from 4 to 28; personal barriers and safety and personal and social value subscales range from 5 to 35.

Figure 1 contains our initial hypothesized path model. Both the TBCRC and PAF models had good model fits with RMSEA of 0.02 and 0.00, respectively (Figure 2). Overall test of equality of regression paths across survey cohorts was conducted and better fit was provided when paths are separated by survey cohorts (Chi‐squared difference = 22.02, degrees of freedom difference = 10, *p* value = 0.015). Though all variables in our model had modest associations with participation in a breast cancer clinical trial (standardized coefficients < 0.30), the most influential factor was attitudes toward clinical trials (TBCRC: standardized direct effect 0.21, *p* < 0.001; PAF: standardized direct effect 0.13, *p* = 0.01; Table 2). Additionally, clinical trial knowledge had a significant association with attitudes toward clinical trials (TBCRC: 0.22, *p* < 0.001; PAF: 0.15, *p* = 0.003). Conversely, the association between clinical trial knowledge and participation differed in the survey cohort models. For the TBCRC model, the association had a significant effect (standardized total effect: 0.27, *p* < 0.001); however, the association was not significant for the PAF model (standardized total effect 0.06, *p* = 0.28). The sensitivity analysis including respondents who reported discussing a clinical trial with their provider were similar to the overall results, with attitudes toward clinical trials being positively associated with enrollment and clinical trial knowledge was associated with attitudes; however knowledge did not have a statistically significant association with trial participation (Table S2).

**TABLE 2:** Model‐estimated standardized total, direct, and indirect path coefficients, standard errors, and *p* values of variables sought to influence breast cancer clinical trial participation by survey cohort.

	Standardized total effects (standard error)	*p*	Standardized direct effects	*p*	Standardized indirect effects (standard error)	*p*
Patient Advocate Foundation, *n* = 358						
Mid‐level of annual household income → ever participated in a breast cancer clinical trial	0.0815 (0.0619)	0.1884	0.0815 (0.0619)	0.1884		
Employed → mid‐level annual household income	0.5254 (0.0631)	**< 0.0001**	0.5254 (0.0631)	**< 0.0001**		
Employed → ever participated in a breast cancer clinical trial	0.1194 (0.0788)	0.0869	0.0766 (0.0853)	0.3690	0.0428 (0.0330)	0.1945
Retired/disabled → mid‐level annual household income	0.1429 (0.0662)	**0.0309**	0.1429 (0.0662)	**0.0309**		
Retired/disabled → ever participated in a breast cancer clinical trial	0.1209 (0.0796)	0.1290	0.1092 (0.0800)	0.1720	0.0116 (0.0104)	0.2617
Years of education → mid‐level annual household income	0.2637 (0.0446)	**< 0.0001**	0.2637 (0.0446)	**< 0.0001**		
Years of education → ever participated in a breast cancer clinical trial	0.0918 (0.0536)	0.0869	0.0559 (0.5750)	0.3314	0.0360 (0.0215)	0.0950
Years of education → knowledge of clinical trials score	0.2475 (0.0497)	**< 0.0001**	0.2475 (0.0497)	**< 0.0001**		
Years of education → Attitudes Toward Cancer Trials Scale	0.0380 (0.0150)	**0.0113**			0.0380 (0.0150)	**0.0113**
Attitudes Toward Cancer Trials Scale → ever participated in a breast cancer clinical trial	0.1280 (0.0519)	**0.0136**	0.1280 (0.0519)	**0.0136**		
Knowledge of clinical trials score → Attitudes Toward Cancer Trials Scale	0.1537 (0.0517)	**0.0029**	0.1537 (0.0517)	**0.0029**		
Knowledge of clinical trials score → ever participated in a breast cancer clinical trial	0.0585 (0.0536)	0.2753	0.0388 (0.0539)	0.4709	0.0197 (0.0104)	0.0586
Early stage breast cancer → ever participated in a breast cancer clinical trial	−0.0932 (0.0568)	0.1010	−0.0932 (0.0568)	0.1010		
Translational Breast Cancer Research Consortium, *n* = 254						
Mid‐level of annual household income → ever participated in a breast cancer clinical trial	−0.0204 (0.0628)	0.7456	−0.0204 (0.0628)	0.7456		
Employed → mid‐level annual household income	0.2019 (0.1036)	0.0513	0.2019 (0.1036)	0.0513		
Employed → ever participated in a breast cancer clinical trial	0.0165 (0.1046)	0.8749	0.0206 (0.1053)	0.8451	−0.0041 (0.0278)	**0.0018**
Retired/disabled → mid‐level annual household income	−0.1334 (0.1025)	0.1930	−0.1334 (0.1025)	0.1930		
Retired/disabled → ever participated in a breast cancer clinical trial	0.0422 (0.1032)	0.6825	0.0395 (0.1035)	0.7028	0.0027 (0.0086)	0.7529
Years of education → mid‐level annual household income	0.1593 (0.0590)	**0.0070**	0.1593 (0.0590)	**0.0070**		
Years of education → ever participated in a breast cancer clinical trial	0.1232 (0.0606)	**0.0454**	0.0366 (0.0640)	0.5676	0.0866 (0.0278)	**0.0018**
Years of education → knowledge of clinical trials score	0.3353 (0.0558)	**< 0.0001**	0.3353 (0.0558)	**< 0.0001**		
Years of education → Attitudes Toward Cancer Trials Scale	0.0753 (0.0240)	**0.0017**			0.0753 (0.0240)	**0.0017**
Attitudes Toward Cancer Trials Scale → ever participated in a breast cancer clinical trial	0.2149 (0.0584)	**0.0002**	0.2149 (0.0584)	**0.0002**		
Knowledge of clinical trials score → Attitudes Toward Cancer Trials Scale	0.2245 (0.0597)	**0.0002**	0.2245 (0.0597)	**0.0002**		
Knowledge of clinical trials score → ever participated in a breast cancer clinical trial	0.2680 (0.0611)	**< 0.0001**	0.2198 (0.0619)	**0.0004**	0.0483 (0.0185)	**0.009**
Early stage breast cancer → ever participated in a breast cancer clinical trial	−0.1543 (0.0606)	**0.0109**	−0.1543 (0.0606)	**0.0109**		

*Note:* Clinical trial knowledge score ranges from 0 to 7 with higher scores indicating more knowledge about clinical trials. Knowledge scores equal to or > 5 were considered high knowledge, as this would be considered a passing grade. Attitudes Toward Cancer Trials Scale ranges from 18 to 126 with higher scores indicating more positive attitudes toward clinical trials. Personal beliefs and trust in the research process subscales range from 4 to 28; personal barriers and safety and personal and social value subscales range from 5 to 35. Bolded values are significant at the alpha level of 0.05.

The exploratory logistic regression model was employed to investigate the interaction between clinical trial knowledge and attitudes on participation in a breast cancer clinical trial. Although we planned to run these models separately, the model for the TBCRC‐only cohort did not converge due to separation of data points, meaning there was not enough variation in the dataset for the model to run and produce an estimate. Therefore, the logistic regression model included both PAF and TBCRC cohorts combined. Of individuals with positive‐leaning attitudes, individuals with high vs. low trial knowledge had 1.78 times the odds of participation in clinical trial (95% CI 1.07–2.98; Table 3). Additionally, among respondents with high knowledge, respondents with positive‐ vs. negative‐leaning attitudes had 8.45 times the odds of participation in clinical trial (95% CI 1.10–64.81). The results from the PAF only survey cohort are presented in Table S3.

**TABLE 3:** Model‐estimated odds ratios and 95% confidence intervals assessing the association between both attitudes toward clinical trials and knowledge about clinical trials and participation in a clinical trial (*N* = 612).

	Odds ratios (95% CI)	Predicted probabilities (95% CI)
Respondents with high knowledge		
Positive‐leaning attitudes	**8.45 (1.10–64.81)**	0.22 (0.17–0.28)
Negative‐leaning attitudes	Reference	0.03 (0.004–0.20)
Respondents with low knowledge		
Positive‐leaning attitudes	1.51 (0.66–3.47)	0.14 (0.10–0.19)
Negative‐leaning attitudes	Reference	0.10 (0.05–0.18)
Respondents with positive‐leaning attitudes		
High knowledge	**1.78 (1.07–2.98)**	0.22 (0.17–0.28)
Low knowledge	Reference	0.14 (0.10–0.19)
Respondents with negative‐leaning attitudes		
High knowledge	0.32 (0.04–2.72)	0.03 (0.004–0.20)
Low knowledge	Reference	0.10 (0.05–0.18)

*Note:* Model controlled for age at diagnosis, race (due to small numbers, categories with less than 20 individuals were grouped into a singular group for modeling purposes), ethnicity, cancer stage, education, employment, income, and survey cohort. Clinical trial knowledge score ranges from 0 to 7 with higher scores indicating more knowledge about clinical trials. Knowledge scores equal to or > 5 were considered high knowledge, as this would be considered a passing grade. Attitudes Toward Cancer Trials Scale ranges from 18 to 126 with higher scores indicating more positive attitudes toward clinical trials. Bolded values are significant at the alpha level of 0.05.

Abbreviation: CI, confidence intervals.

### 3.3 Attitudes Toward Clinical Trials and Patient‐Level Demographics

For further exploration, we modeled the association between patient‐level demographics and attitudes toward breast cancer trials. Compared to those with low knowledge, respondents with high clinical trial knowledge had two times the odds of having positive‐leaning attitudes (OR 2.10, 95% CI 1.26–3.50; Table 4). The results from the PAF‐only survey cohort are presented in Table S4. Cohorts were combined for this model since the TBCRC‐only model did not converge due to separation of data points, as above.

**TABLE 4:** Model‐estimated odds ratios and 95% confidence intervals exploring the association between patient‐level demographics and positive‐leaning attitudes toward breast cancer clinical trials (*N* = 612).

	Odds ratios (95% CI)	Predicted probabilities (95% CI)
Knowledge of clinical trials		
High knowledge	**2.10 (1.26–3.50)**	0.90 (0.85–0.93)
Low knowledge	Reference	0.81 (0.75–0.85)
Education level		
Bachelor's degree or higher	0.66 (0.39–1.12)	0.84 (0.79–0.88)
High school diploma or lower	0.68 (0.35–1.30)	0.84 (0.74–0.91)
Some college	Reference	0.89 (0.83–0.92)
Annual household income		
$50,000 or more	1.41 (0.81–2.46)	0.87 (0.83–0.91)
Less than $50,000	Reference	0.83 (0.77–0.88)
Breast cancer stage		
Early	0.83 (0.51–1.35)	0.84 (0.79–0.88)
Late	Reference	0.87 (0.82–0.90)
Employment status		
Employed	0.65 (0.30–1.42)	0.85 (0.80–0.90)
Retired/disabled	0.60 (0.28–1.26)	0.84 (0.79–0.89)
Unemployed/other	Reference	0.90 (0.82–0.95)
Age at diagnosis		
49 year or younger	1.13 (0.71–1.81)	0.86 (0.82–0.90)
50 or older	Reference	0.85 (0.79–0.89)
Race		
Black	0.74 (0.43–1.27)	0.82 (0.74–0.89)
Other	0.73 (0.34–1.57)	0.82 (0.69–0.91)
White	Reference	0.86 (0.83–0.90)
Hispanic or Latino(a) origin or descent		
No	0.71 (0.31–1.62)	0.85 (0.81–0.88)
Yes	Reference	0.89 (0.78–0.95)
Survey cohort		
Patient Advocate Foundation	**0.33 (0.18–0.63)**	0.79 (0.73–0.83)
Translational Breast Cancer Research Consortium	Reference	0.92 (0.87–0.95)

*Note:* Clinical trial knowledge score ranges from 0 to 7 with higher scores indicating more knowledge about clinical trials. Knowledge scores equal to or > 5 were considered high knowledge, as this would be considered a passing grade. Attitudes Toward Cancer Trials Scale ranges from 18 to 126 with higher scores indicating more positive attitudes toward clinical trials. Bolded values are significant at the alpha level of 0.05.

Abbreviation: CI, confidence intervals.

### 3.4 Knowledge of Clinical Trials and Patient‐Level Demographics

We also modeled the association between patient‐level demographics and clinical trial knowledge among the entire sample. We found that individuals with positive‐ vs. negative‐leaning attitudes had two times the odds of having high knowledge (OR 2.11, 95% CI 1.26–3.51; Table S5). Furthermore, we found that respondents with a Bachelor's degree or higher vs. some college and < 50 vs. ≥ 50 had higher odds of clinical trial knowledge (OR 2.09, 95% CI 1.37–3.19; OR 1.55, 95% CI 1.05–2.29, respectively). Finally, we found that, when compared to White respondents, Black respondents had lower odds of having high knowledge (OR 0.40, 95% CI 0.23–0.70).

## 4 Discussion

This study includes a unique set of data containing socioeconomic demographics, attitudes, knowledge, and previous participation in breast cancer clinical trials among two survey cohorts of women with previous breast cancer diagnoses. We found that positive clinical trial attitudes were associated with breast cancer clinical trial participation. Although there are many behavioral interventions that utilize attitudes to spur behavior change, there is a dearth of research that examines patients' attitudes toward cancer clinical trials. In a 2020 study assessing attitudes toward clinical trials, Lewin and colleagues found that adolescents and young adult (AYAs) with cancer, who typically have the lowest participation in cancer clinical trials, viewed clinical trials less favorably than their older counterparts. AYAs reported trials to be unsafe and more difficult, potentially due to their views of more personal barriers to trial participation [21]. Research has also assessed public and providers' attitudes toward clinical trials. In late 2020, Wong and colleagues found, through qualitative interviews, that community oncologists felt that patients' attitudes affected their trial participation [22]. More research is needed to better understand how attitudes toward clinical trials are associated with cancer clinical trial participation.

Our study also showed that knowledge was a strong predictor of clinical trial participation overall. With the cohort‐specific models, the TBCRC survey cohort had an association between knowledge and trial participation; however, we did not find the same for the PAF survey cohort. Therefore, more research is needed to understand if knowledge could be a modifiable factor for underrepresented patient populations, or if another factor is more imperative to this patient population. For example, Virani and colleagues found that a third of rural patients with cancer (who are underrepresented in trials) had never heard about a clinical trial and that among those who did not participate, 35% said it was due to lack of information about trials [23]. However, there is limited research on the best strategies to deliver knowledge of cancer clinical trials to patients. A study by Jacobsen and colleagues found that a psychoeducational intervention versus printed educational material increased both willingness to participate in and positive attitudes toward clinical trials [24]. This is in concordance with our results that attitudes and knowledge are associated with one another and clinical trial participation: respondents with both positive attitudes and high knowledge had higher participation percentages. However, more than one strategy to increase knowledge and attitudes may be needed depending on the particular patient population. Prior research has shown that older adults [25] and rural residents [26] face more digital health literacy challenges. Furthermore, individuals in rural or disadvantaged areas face barriers to internet access [27]. These may affect the efficacy of given interventions as research is incorporating more technology, including the use of the online consent forms and questionnaires related to the research study plus the inclusion of telemedicine visits with staff and providers conducting the trial. Furthermore, the use of the four competencies used to enable an individual to navigate the health continuum—access health information, understand and comprehend health information, appraise and evaluate the found health information, and apply and use the health information to make a decision [28]—could be utilized in future research to increase knowledge and attitudes toward clinical trials.

We did not find any patient‐level socioeconomic demographics nor clinical characteristics to be associated with more positive attitudes to clinical trials, as the majority of individuals in our study had positive‐leaning attitudes toward clinical trials. However, we expected to find different levels of attitudes toward clinical trials, specifically for Black or African American individuals, as the historical mistreatment of Black patients has created a level of mistrust in the medical community [29]. However, there are multiple factors that influence attitudes that we were unable to capture in this study, such as experiences, beliefs, feelings, norms, influences [30]. However, we did find some patient‐level demographics to be associated with high knowledge of clinical trials, such as age, education, and race. Although individuals who were younger and had higher education levels had higher odds of having high knowledge, we found that Black individuals had lower odds of having high knowledge. A systematic review by Rivers and colleagues that looked at why Black patients are underrepresented in clinical trials found that negative attitudes and low knowledge are common among Black patients and are associated with lower likelihood of participating in a clinical trial [31]. Authors also found that community perception of trials was one of the strongest factors that influenced Black patients to enroll in a trial. Overall, future research is needed to understand the connections between both patient‐level attitudes and knowledge with the importance of and reasons for participating in a clinical trial, the logistical, financial, and societal environment patients live in that facilitates participation, and the habits of those who have and have not participated in a clinical trial. Understanding these factors via more robust study designs and qualitative interviews will be important in creating future behavioral interventions focused on increasing cancer clinical trial participation and representation.

Although patients' knowledge and attitudes appear to influence their cancer clinical participation, patients must first be offered a clinical trial in order to participate [3]. Another important finding from our study was that almost two in three individuals reported that a provider did not discuss a breast cancer clinical trial with them. However, NCCN guidelines state that clinical trials are standard of care and discussions are recommended [32]; therefore, providers should discuss clinical trials as a treatment option with patients. Furthermore, cancer can cause distress in patients [33, 34], and patients may choose to rely heavily on their provider for guidance in choosing to participate in trial or not [35, 36]. Therefore, providers are essential to their patients' participation in clinical trials; they should make sure clinical trial discussions are distinct and clear, offering patients the risks and benefits of clinical trial participation.

This study should be considered in light of several limitations. This sample of two nationwide cohorts may not reflect the entire population of patients with breast cancer. Additionally, the timing of these surveys was during the COVID‐19 pandemic. As this was a cross sectional study, causality cannot be inferred from results. Thus, we are unaware if clinical trial attitudes and knowledge changed after participating in a trial or if attitudes and knowledge were enabling factors for participation. Additionally, respondents who were recruited via TBCRC were done via social media by breast cancer advocacy groups and TBCRC investigators; therefore, selection bias may be present. Furthermore, recall bias from respondents may be present. We were unable to ascertain if a trial was available or offered to respondents. An additional bias is the temporal issue among the different time points of when patients may have participated in a trial and the demographics and characteristics they reported at the time of the survey. Income levels, insurance coverage, or employment status may have differed at the time of respondents' clinical trial participation versus when they completed the survey. Finally, information about the region of respondent residence, place of residence, and provider involvement was not captured, as these may be associated with access to and participation in clinical trials.

## 5 Conclusions

In this study of two survey cohorts of women with breast cancer typically represented and underrepresented in cancer clinical trials, we found positive attitudes toward cancer clinical trials were associated with breast cancer clinical participation, whether that be prior to or following participation in a breast cancer clinical trial. We found that high knowledge of trials was associated with positive‐leaning attitudes, but no other patient‐level demographics. Therefore, interventions to increase clinical trial participation should focus on increasing both knowledge and attitudes.

## Author Contributions

Study concept/design: N.E.C., C.P.W., E.B.L., A.A., R.G., S.B.W., G.B.R. Provision of study material or patients: K.G., K.L.S., A.C.W. Data collection/assembly: K.G., K.L.S., A.C.W. Data analysis and interpretation: All authors. Manuscript writing: All authors. Final approval of manuscript: All authors.

## Conflicts of Interest

Dr. Caston is supported by the National Cancer Institute's National Research Service Award sponsored by the Lineberger Comprehensive Cancer Center at the University of North Carolina at Chapel Hill (T32 CA116339). Dr. Rocque received research funding from Genentech, Pfizer, Daiichi Sankyo and Armada and consulting fees for Genentech and Pfizer. Dr. Levitan received research funding from Amgen Inc. and fees for serving on a data safety and monitoring board from University of Pittsburgh. Dr. Wheeler has received funding from The Pfizer Foundation and AstraZeneca. Dr. Smith has received funding from Pfizer. Dr Smith's family member has stock in Abbvie and Abbott Labs. Dr. Smith has been employed by AstraZeneca and is currently employed by Merck; she currently has stock in Merck. Dr. Smith contributed to this work while she was at her previous place of employment, Johns Hopkins University. Dr. Melisko has received research/clinical trial funding from Novartis, Puma, KCRN research, OBI Pharma, and Seagen.

## Supporting information


Data S1

## Data Availability

The data that support the findings of this study are available from the Translational Breast Cancer Research Consortium and Patient Advocate Foundation upon reasonable request.
