# Supplementary material for: Understanding Associations Between Patient‐Level Factors and Participation in a Breast Cancer Clinical Trial
Source: Cancer Med. 2025 Aug 20;14(16):e71010. doi: 10.1002/cam4.71010 (PMC12367865; doi:10.1002/cam4.71010)
Supplement: Supplementary file 1 — Data S1 [file CAM4-14-e71010-s001.docx]

Supplemental Index

1. Methods
2. Results
3. Tables
4. Figure

Supplemental Methods

Sample Size Considerations: For path models, appropriateness of a sample size can be examined using the RMSEA (Root Mean Squared Error of Approximation) index. Typically, values of RMSEA smaller than 0.06 indicate good model fit, values of RMSEA between 0.06 and 0.1 indicate fair fit, and values of RMSEA greater than 0.1 indicate poor model fit.^19^ A transformation of the RMSEA estimate follows approximately a non-central chi-squared distribution.^20^ Using this sampling distribution, a test of whether the RMSEA estimate is smaller than 0.1 (i.e. a test of the null hypothesis that a model provides poor fit to the data) can be conducted. For the model in Figure 1, there are five continuous variables, three binary employment status indicators, and two cancer stage indicators; therefore with ten observed variables and indicators, the number of unique elements of the covariance matrix is 55. In the model, there are ten variance/error terms and 14 paths, so the number of degrees of freedom is 55-10-14=31. Using the software by MacCallum et al.^37^, assuming a null RMSEA of 0.1, an alternative RMSEA of 0.06, model df=31, 80% power, and alpha=0.05, the sample necessary to reject ‘poor model fit’ given an observed RMSEA of 0.06, is N=182. Therefore, the expected sample of N=619 is well powered to address this aim and conduct multiple group analyses.

Age at diagnosis: The cutoff of 50 years of age at diagnosis was used due to the following clinical reasons: typical age when mammography starts and individuals who are diagnosed under the age of 50 typically have a familial history of breast cancer. Furthermore, as far as the data structure were concerned, splitting it at 50 years of age was also about the median cutoff for this variable.

Additional statistical analyses: To determine the internal reliability for the Attitudes Toward Cancer Trials Scale, we ran a Cronbach’s alpha, where a “good” coefficient ranges between 0.65-0.80.^38^

Results

*Cronbach’s alpha:* We found a Cronbach’s alpha of 0.62 for the Attitudes Toward Cancer Trials Scale.

Supplemental Table 1. Patient demographics and clinical characteristics by survey cohort: Patient Advocate Foundation (n=358) or Translational Breast Cancer Research Consortium (n=254).

|  | Patient Advocate Foundation sample  n=358 | Translational Breast Cancer Research Consortium sample n=254 | Cramer's V or Cohen's d |
| --- | --- | --- | --- |
| **Age at diagnosis, in years** |  |  | 0.09 |
| 49 or younger | 186 (52) | 156 (61) |  |
| 50 or older | 172 (48) | 98 (39) |  |
| **Race** |  |  | 0.37 |
| American Indian/Alaska Native | 1 (0) | 0 (0) |  |
| Asian | 10 (3) | 3 (1) |  |
| Black/African American | 102 (28) | 7 (3) |  |
| More than one race | 11 (3) | 7 (3) |  |
| Native Hawaiian or Pacific Islander | 1 (0) | 0 (0) |  |
| Other | 16 (4) | 3 (1) |  |
| White/Caucasian | 217 (61) | 234 (92) |  |
| **Ethnicity** |  |  | 0.12 |
| Hispanic or Latino(a) origin or descent | 39 (11) | 11 (4) |  |
| Non-Hispanic or Latino(a) origin or descent | 319 (89) | 243 (96) |  |
| **Education level** |  |  | 0.43 |
| High school or less | 65 (18) | 7 (3) |  |
| Some college | 157 (44) | 43 (17) |  |
| Bachelor's degree or more | 136 (38) | 204 (80) |  |
| **Annual household income** |  |  | 0.52 |
| Less than $50,000 | 248 (69) | 43 (17) |  |
| $50,000 or more | 110 (31) | 211 (83) |  |
| **Employment status** |  |  | 0.17 |
| Employed | 132 (37) | 136 (54) |  |
| Retired/disabled | 175 (49) | 94 (37) |  |
| Unemployed/Other | 51 (14) | 24 (9) |  |
| **Breast cancer stage** |  |  | 0.04 |
| Early | 191 (53) | 124 (49) |  |
| Late | 167 (47) | 130 (51) |  |
| **Years since cancer diagnosis** |  |  | 0.05 |
| Less than 5 years ago | 261 (73) | 175 (69) |  |
| 5-10 years ago | 49 (14) | 42 (17) |  |
| More than 10 years ago | 48 (13) | 37 (15) |  |
| **Insurance** |  |  | 0.36 |
| Ever Medicaid | 95 (27) | 12 (5) |  |
| Ever Medicare | 114 (32) | 53 (21) |  |
| None/other/unknown | 24 (7) | 18 (7) |  |
| Private | 125 (35) | 171 (67) |  |
| **Ever participated in a breast cancer clinical trial** |  |  | 0.08 |
| Yes | 54 (15) | 55 (22) |  |
| No | 304 (85) | 199 (78) |  |

SD=standard deviation.

Supplemental Table 2. Sensitivity analysis: Model-estimated standardized total, direct, and indirect path coefficients, standard errors, and p-values of variables sought to influence breast cancer clinical trial participation among respondents who reported discussing a clinical trial with their breast cancer provider (n=218).

|  | Standardized total effects (standard error) | p-value | Standardized direct effects | p-value | Standardized indirect effects (standard error) | p-value |
| --- | --- | --- | --- | --- | --- | --- |
| Mid-level of annual household income —> ever participated in a breast cancer clinical trial | -0.0157 (0.0816) | 0.8474 | -0.0157 (0.0816) | 0.8474 |  |  |
| Employed —> mid-level annual household income | 0.2398 (0.0972) | **0.0136** | 0.2398 (0.0972) | **0.0136** |  |  |
| Employed —> ever participated in a breast cancer clinical trial | 0.0062 (0.1181) | 0.9581 | 0.0109 (0.1197) | 0.9336 | -0.0038 (0.0196) | 0.8479 |
| Retired/disabled —> mid-level annual household income | -0.0928 (0.0955) | 0.3311 | -0.0928 (0.0955) | 0.3311 |  |  |
| Retired/disabled —> ever participated in a breast cancer clinical trial | 0.0878 (0.1150) | 0.4453 | 0.0863 (0.1152) | 0.4538 | 0.0015 (0.0077) | 0.8503 |
| Years of education —> mid-level annual household income | 0.4215 (0.0531) | **<0.0001** | 0.4215 (0.0531) | **<0.0001** |  |  |
| Years of education —> ever participated in a breast cancer clinical trial | 0.0362 (0.0694) | 0.6022 | -0.0116 (0.0819) | 0.8871 | 0.0478 (0.0458) | 0.2961 |
| Years of education —> knowledge of clinical trials score | 0.4004 (0.0570) | **<0.0001** | 0.4004 (0.0570) | **<0.0001** |  |  |
| Years of education —> Attitudes Toward Cancer Trials Scale | 0.1323 (0.0316) | **<0.0001** |  |  | 0.1323 (0.0316) | **<0.0001** |
| Attitudes Toward Cancer Trials Scale —> ever participated in a breast cancer clinical trial | 0.2358 (0.0675) | **0.0005** | 0.2358 (0.0675) | **0.0005** |  |  |
| Knowledge of clinical trials score —> Attitudes Toward Cancer Trials Scale | 0.3305 (0.0605) | **<0.0001** | 0.3305 (0.0605) | **<0.0001** |  |  |
| Knowledge of clinical trials score —> ever participated in a breast cancer clinical trial | 0.1360 (0.0723) | 0.0599 | 0.0580 (0.0747) | 0.4373 | 0.0779 (0.0269) | **0.0037** |
| Early stage breast cancer —> ever participated in a breast cancer clinical trial | -0.0440 (0.0675) | 0.5147 | -0.0440 (0.0675) | 0.5147 |  |  |

Clinical trial knowledge score ranges from 0-7 with higher scores indicating more knowledge about clinical trials. Knowledge scores equal to or greater than 5 were considered high knowledge, as this would be considered a passing grade. Attitudes Toward Cancer Trials Scale ranges from 18-126 with higher scores indicating more positive attitudes toward clinical trials. Personal beliefs and trust in the research process subscales range from 4-28; personal barriers and safety and personal and social value subscales range from 5-35. Bolded values are significant at the alpha level of 0.05.

Supplemental Table 3. Model-estimated odds ratios and 95% confidence intervals assessing the association between both attitudes toward clinical trials and knowledge about clinical trials and participation in a clinical trial for the Patient Advocate Foundation survey cohort (N=358).

|  | Odds ratios  (95% CI) | Predicted probabilities (95% CI) |
| --- | --- | --- |
| **Respondents with high knowledge** |  |  |
| Positive-leaning attitudes | 4.05 (0.50-32.99) | 0.15 (0.09-0.24) |
| Negative-leaning attitudes | Reference | 0.04 (0.01-0.25) |
| **Respondents with low knowledge** |  |  |
| Positive-leaning attitudes | 1.42 (0.60-3.35) | 0.15 (0.10-0.21) |
| Negative-leaning attitudes | Reference | 0.11 (0.05-0.21) |
| **Respondents with positive-leaning attitudes** |  |  |
| High knowledge | 1.04 (0.51-2.10) | 0.15 (0.09-0.24) |
| Low knowledge | Reference | 0.15 (0.10-0.21) |
| **Respondents with negative-leaning attitudes** |  |  |
| High knowledge | 0.36 (0.04-3.16) | 0.04 (0.01-0.25) |
| Low knowledge | Reference | 0.11 (0.05-0.21) |

Model controlled for age at diagnosis, race (due to small numbers, categories with less than 20 individuals were grouped into a singular group for modeling purposes), ethnicity, cancer stage, education, employment, and income. Clinical trial knowledge score ranges from 0-7 with higher scores indicating more knowledge about clinical trials. Knowledge scores equal to or greater than 5 were considered high knowledge, as this would be considered a passing grade. Attitudes Toward Cancer Trials Scale ranges from 18-126 with higher scores indicating more positive attitudes toward clinical trials. Bolded values are significant at the alpha level of 0.05. CI=confidence intervals. Model for Translational Breast Cancer Research Consortium cohort did not converge due to separation of data points.

Supplemental Table 4. Model-estimated odds ratios and 95% confidence intervals exploring the association between patient-level demographics and positive-leaning attitudes toward breast cancer clinical trials among Patient Advocate Foundation survey respondents (N=358).

|  | Odds ratios  (95% CI) | Predicted probabilities  (95% CI) |
| --- | --- | --- |
| **Knowledge of clinical trials** |  |  |
| High knowledge | 1.56 (0.89-2.75) | 0.81 (0.72-0.87) |
| Low knowledge | Reference | 0.73 (0.66-0.78) |
| **Education level** |  |  |
| Bachelor's degree or higher | 0.72 (0.40-1.28) | 0.73 (0.64-0.80) |
| High school diploma or lower | 0.62 (0.32-1.20) | 0.70 (0.57-0.80) |
| Some college | Reference | 0.79 (0.72-0.85) |
| **Annual household income** |  |  |
| $50,000 or more | 1.38 (0.75-2.56) | 0.79 (0.70-0.86) |
| Less than $50,000 | Reference | 0.73 (0.67-0.79) |
| **Breast cancer stage** |  |  |
| Early | 0.80 (0.46-1.38) | 0.73 (0.66-0.80) |
| Late | Reference | 0.78 (0.70-0.84) |
| **Employment status** |  |  |
| Employed | 0.69 (0.30-1.57) | 0.74 (0.65-0.82) |
| Retired/disabled | 0.71 (0.33-1.55) | 0.75 (0.67-0.81) |
| Unemployed/other | Reference | 0.81 (0.68-0.89) |
| **Age at diagnosis** |  |  |
| 49 year or younger | 1.05 (0.62-1.77) | 0.76 (0.69-0.82) |
| 50 or older | Reference | 0.75 (0.67-0.81) |
| **Race** |  |  |
| Black | 0.74 (0.42-1.29) | 0.72 (0.62-0.80) |
| Other | 0.71 (0.32-1.59) | 0.71 (0.54-0.84) |
| White | Reference | 0.78 (0.71-0.83) |
| **Hispanic or Latino(a) origin or descent** |  |  |
| No | 0.67 (0.28-1.60) | 0.75 (0.69-0.79) |
| Yes | Reference | 0.81 (0.66-0.91) |

Bolded values are significant at the alpha level of 0.05. CI=confidence intervals. Model for Translational Breast Cancer Research Consortium cohort did not converge due to separation of data points.

Supplemental Table 5. Model-estimated odds ratios and 95% confidence intervals exploring the association between patient-level demographics and high knowledge of breast cancer clinical trials (N=612).

|  | Odds ratios (95% CI) | Predicted probabilities (95% CI) |
| --- | --- | --- |
| **Attitudes toward clinical trials** |  |  |
| Positive-leaning attitudes | **2.11 (1.26-3.51)** | 0.50 (0.45-0.55) |
| Negative-leaning attitudes | Reference | 0.32 (0.23-0.43) |
| **Education level** |  |  |
| Bachelor's degree or higher | **2.09 (1.37-3.19)** | 0.55 (0.49-0.61) |
| High school diploma or lower | 0.77 (0.39-1.51) | 0.31 (0.20-0.46) |
| Some college | Reference | 0.37 (0.30-0.45) |
| **Annual household income** |  |  |
| $50,000 or more | 1.20 (0.82-1.81) | 0.49 (0.42-0.56) |
| Less than $50,000 | Reference | 0.44 (0.37-0.51) |
| **Breast cancer stage** |  |  |
| Early | 1.22 (0.82-1.81) | 0.49 (0.42-0.55) |
| Late | Reference | 0.44 (0.37-0.51) |
| **Employment status** |  |  |
| Employed | 0.62 (0.33-1.15) | 0.42 (0.35-0.50) |
| Retired/disabled | 0.78 (0.43-1.42) | 0.48 (0.41-0.55) |
| Unemployed/other | Reference | 0.54 (0.41-0.67) |
| **Age at diagnosis** |  |  |
| 49 year or younger | **1.55 (1.05-2.29)** | 0.51 (0.45-0.57) |
| 50 or older | Reference | 0.40 (0.34-0.47) |
| **Race** |  |  |
| Black | **0.40 (0.23-0.70)** | 0.30 (0.20-0.41) |
| Other | 0.66 (0.34-1.29) | 0.41 (0.27-0.57) |
| White | Reference | 0.51 (0.46-0.56) |
| **Hispanic or Latino(a) origin or descent** |  |  |
| No | 1.87 (0.91-3.83) | 0.48 (0.43-0.52) |
| Yes | Reference | 0.33 (0.20-0.49) |
| **Survey cohort** |  |  |
| Patient Advocate Foundation | **0.43 (0.28-0.67)** | 0.38 (0.32-0.44) |
| Translational Breast Cancer Research Consortium | Reference | 0.59 (0.51-0.66) |

Clinical trial knowledge score ranges from 0-7 with higher scores indicating more knowledge about clinical trials. Knowledge scores equal to or greater than 5 were considered high knowledge, as this would be considered a passing grade. Attitudes Toward Cancer Trials Scale ranges from 18-126 with higher scores indicating more positive attitudes toward clinical trials. Bolded values are significant at the alpha level of 0.05. CI=confidence intervals.

Supplemental Figure. Survey questions.

You are being asked to complete an online research survey from [Patient Advocate Foundation or Translational Breast Cancer Research Consortium]. We are conducting this research because we want to learn how people feel about participating in breast cancer clinical trials.

Breast cancer clinical trials are a type of research done to learn about breast cancer and about how to improve breast cancer treatments. Your responses to this survey will build on prior research about barriers to clinical trial participation and may help breast cancer researchers improve how they conduct breast cancer clinical trials during and after the pandemic.

This survey was deemed exempt by the University of Alabama at Birmingham Institutional Review Board. Completing this survey is optional and your answers will be kept anonymous. By beginning this survey, you are providing consent to participate in this research.

This survey will take approximately 15 minutes of your time. Please mark only one answer for each question unless otherwise indicated.
  
*For the Patient Advocate Foundation survey only: Individuals who complete the entire survey will be entered into a drawing to win one of five digital $100 Amazon gift cards. Winners will be notified via email by Patient Advocate Foundation.*

1. I agree to participate in this survey.

- Yes
- No

1. Have you ever been diagnosed with breast cancer?

- Yes
- No

1. How many years ago were you first diagnosed with breast cancer?

- Less than 1 year ago
- 1-5 years ago
- 5-10 years ago
- More than 10 years ago

1. Which of the following statements most accurately describes you?

- I have been diagnosed with early stage breast cancer (breast cancer involving the breast with or without involving the armpit lymph glands)
- I have been diagnosed with metastatic breast cancer (breast cancer that has spread to other parts of the body)
- Other

1. Are you at least 18 years of age?

- Yes
- No

1. Have you ever participated in a breast cancer clinical trial?

- Yes
- No

1. Are you currently participating in a breast cancer clinical trial?

- Yes
- No

1. Has a breast cancer provider ever discussed a breast cancer clinical trial with you?

- Yes
- No

1. For the next questions, please mark the number indicating how much you agree or disagree with each of the statements about clinical trials:

| 1 = Strongly Disagree 7 = Strongly Agree | 1 | 2 | 3 | 4 | 5 | 6 | 7 |
| --- | --- | --- | --- | --- | --- | --- | --- |
| I’d get improved cancer treatment if I took part in a clinical trial |  |  |  |  |  |  |  |
| People who join clinical trials have a better chance of beating their cancer |  |  |  |  |  |  |  |
| Joining a clinical trial would mean I’d receive the best existing cancer treatment |  |  |  |  |  |  |  |
| By joining a clinical trial, I would receive better health care |  |  |  |  |  |  |  |
| Taking part in a clinical trial is a lot more trouble than just getting the usual treatment |  |  |  |  |  |  |  |
| Getting treatment in a clinical trial is less safe than getting the usual cancer treatment |  |  |  |  |  |  |  |
| Treatments received in a clinical trial could be unsafe for myself |  |  |  |  |  |  |  |
| My taking part in a clinical trial could lead to more health problems |  |  |  |  |  |  |  |
| Joining a clinical trial would make cancer treatment more difficult |  |  |  |  |  |  |  |
| In general, people should know more about clinical trials |  |  |  |  |  |  |  |
| Clinical trials are of little importance to me |  |  |  |  |  |  |  |
| Access to cancer treatment clinical trials is important to me |  |  |  |  |  |  |  |
| People who take part in clinical trials are helping all of us fight cancer |  |  |  |  |  |  |  |
| I feel certain my safety would be watched closely in a clinical trial |  |  |  |  |  |  |  |
| Doctors and nurses tell patients the truth about what to expect during a clinical trial |  |  |  |  |  |  |  |
| If I took part in a clinical, I would be treated like a guinea pig |  |  |  |  |  |  |  |
| Doctors and nurses mislead their patients who are involved in clinical trials |  |  |  |  |  |  |  |
| It would be safe for me to join a clinical trial for treatment |  |  |  |  |  |  |  |

1. Many people have not had any experience with clinical trials before. Please indicate whether you think the following statements about clinical trials are true or not.

|  | True  (1) | False  (2) | Don't know (3) |
| --- | --- | --- | --- |
| In a randomized clinical trial, the treatment you get is decided by chance |  |  |  |
| Clinical trials are only used when standard treatments have not worked |  |  |  |
| Clinical trials test treatments which nobody knows anything about |  |  |  |
| Randomized clinical trials are the best way to find out whether one treatment is better than another |  |  |  |
| Clinical trials are not appropriate for serious diseases like cancer |  |  |  |
| My doctor would know which treatment in a clinical trial was better |  |  |  |
| My doctor would make sure I got the better treatment in a clinical trial |  |  |  |

1. What is your race? Check all that apply.

- American Indian/Alaska Native
- Asian
- Black/African American
- Native Hawaiian or Other Pacific Islander
- White/Caucasian
- Other

1. Are you of Hispanic or Latino(a) origin or descent?

- Yes
- No

1. Please indicate your highest level of academic achievement:

- Less than a high school diploma or equivalent
- High school diploma or equivalent
- Some college, no degree
- Associate’s degree
- Bachelor’s degree
- Master’s or doctoral degree

1. Please indicate your current household income per year in US dollars.

- Less than $20,000
- $20,000 to $34,999
- $35,000 to $49,999
- $50,000 to $74,999
- $75,000 to $99,999
- More than $100,000

1. What is your current employment status?
   Please select what you consider to be your main activity.

- Working full time (≥32 hours/week)
- Working part time (1-31 hours/week)
- Unemployed, looking for work
- Unemployed, not looking for work
- In job training
- Temporarily laid off (no pay)
- Retired
- On short term disability
- On long term disability
- Permanently disabled
- I do not work
- In school
- Other

1. Please indicate your health insurance type.
   Pease select all that apply.

- Private (e.g. BlueCross, Cigna)
- Medicare
- Medicaid
- Tri-Care/Other Military
- Indian Health Service
- The Veterans Health Administration (VA)
- None
- Other

1. How old were you when you were first diagnosed with breast cancer?

- Under 18
- 18-29
- 30-39
- 40-49
- 50-59
- 60-69
- 70 or older
